# The nucleoside antiviral prodrug remdesivir in treating COVID-19 and beyond with interspecies significance

**DOI:** 10.1186/s44149-021-00017-5

**Published:** 2021-09-07

**Authors:** Daisy Yan, One Hyuk Ra, Bingfang Yan

**Affiliations:** 1 Sidney Kimmel Medical College, Thomas Jefferson University, 1025 Walnut St, Philadelphia, PA 19107 USA; 2 Department of Anesthesiology, Brigham and Women’s Hospital, 75 Francis St, Boston, MA 02115 USA; 3 Division of Pharmaceutical Sciences, James L. Winkle College of Pharmacy, University of Cincinnati, Cincinnati, OH 45229 USA

**Keywords:** Animal model, Carboxylesterases, COVID-19, Coronavirus, Drug-drug interactions, interspecies difference, Pandemic, Remdesivir, SARS-CoV-2

## Abstract

Infectious pandemics result in hundreds and millions of deaths, notable examples of the Spanish Flu, the Black Death and smallpox. The current pandemic, caused by SARS-CoV-2 (severe acute respiratory syndrome coronavirus 2), is unprecedented even in the historical term of pandemics. The unprecedentedness is featured by multiple surges, rapid identification of therapeutic options and accelerated development of vaccines. Remdesivir, originally developed for Ebola viral disease, is the first treatment of COVID-19 (Coronavirus disease 2019) approved by the United States Food and Drug Administration. As demonstrated by *in vitro* and preclinical studies, this therapeutic agent is highly potent with a broad spectrum activity against viruses from as many as seven families even cross species. However, randomized controlled trials have failed to confirm the efficacy and safety. Remdesivir improves some clinical signs but not critical parameters such as mortality. This antiviral agent is an ester/phosphorylation prodrug and excessive hydrolysis which increases cellular toxicity. Remdesivir is given intravenously, leading to concentration spikes and likely increasing the potential of hydrolysis-based toxicity. This review has proposed a conceptual framework for improving its efficacy and minimizing toxicity not only for the COVID-19 pandemic but also for future ones caused by remdesivir-sensitive viruses.

## Introduction

Good health is one of the most desirable, and probably the most manageable asset for human being. For most people, good health can be achieved through life-style monitoring, enhanced physical exercises, and understanding of the family history (Abu-Zeinah and DeSancho 2020; Feng et al. 2020; Ford et al. 2020; Nieman and Pence 2020; Sepandi et al. 2020). Infectious pandemics, on the other hand, directly challenge this assumption. Infectious pandemics are defined as outbreaks of infectious diseases over large areas among all populations, although health status may somewhat determine the severity (McGrath et al. 2020; Morens and Fauci 2020; Ransing et al. 2020). The history of infectious pandemics in terms of transmission is tied inextricably to humankind development or activities in a broad sense including society-driven industrialization, increased community engagement and intensified congregations for population-based activities (Coccia 2020; Habersaat et al. 2020; Levin 2020; Moreno et al. 2021). The last contributing factor is relevant, particularly to the current COVID-19 pandemic. Nevertheless, infectious pandemics, even in today’s world, would decimate human populations or cause mankind disruptions tremendously.

Throughout the human history, there have been quite a few infectious pandemics (Glatter and Finkelman 2021; Jester et al. 2018; Johnson and Mueller 2002; Lindenbaum et al. 1967; Pollitzer 1954; Siddique et al. 1995). The brutal killers are exemplified by the Spanish Flu, the Black Death, smallpox and Asiatic cholera. The smallpox is estimated to have killed between 300 and 500 million (Blower and Bernoulli 2004; Krylova and Earn 2020; Thèves et al. 2014). From 1346 to 1353, the Black Death (also called the bubonic plague) caused by the plague bacterium *Y. pestis* (probably other variants) killed 75–200 million people throughout the Asia, Europe, and Africa continents (Glatter and Finkelman 2021). Some infectious pandemics last for decades. For example, the current HIV/AIDS pandemic which started four decades ago, has killed 38 million people worldwide (de Cock et al. 2021; GBD 2017; 22, 23). Among all infectious pathogens, influenza virus is recognized to cause the most pandemics in the last century or so. The 1918 Flu Pandemic killed 20-50 million people, the 1956–58 Asian Flu killed 2 million and the 1968 Flu Pandemic killed 1–4 million people (Jester et al. 2018; Jester et al. 2020; Johnson and Mueller 2002; Morse et al. 2012; Salzberger et al. 2018).

Since the turn of the twenty-first century, the pandemic pathogens have shifted from Influenza viruses to coronaviruses in the sense of pandemic frequency. Indeed, the 2009 H1N1 influenza pandemic, commonly referred to as Swine Flu, is the only Flu pandemic in the twenty-first century (Staniland and Smith 2013). The Flu pandemic of 2009 was initially seen in Mexico and killed approximately 300,000 people worldwide (Staniland and Smith 2013). In contrast, there have three pandemics caused by coronaviruses during the first 21 years of this century. The SARS-CoV pandemic of 2002 (severe acute respiratory syndrome-associated coronavirus) has a confirmed number of over 8000 cases with estimated 813 mortalities (Anderson et al. 2004; Hui and Zumla 2019). The MERS-CoV pandemic of 2012 (middle east respiratory syndrome coronavirus) has much lower number of confirmed cases (~ 2500) but with a similar number of mortalities (858), representing a mortality rate of > 35% (Azhar et al. 2019; Chafekar and Fielding 2018).

The SARS-CoV-2 pandemic of 2019 (severe acute respiratory syndrome-associated coronavirus-2) was reported initially in December of 2019 (Hu et al. 2021). A year and half later, the confirmed cases have reached the number of 203 million with a total mortality of 4.3 million worldwide (Johns Hopkins University Coronavirus Resource Center, 2021). Clearly the number of the fatality has been the greatest since the 1918 Flu Pandemic (Jester et al. 2018; Jester et al. 2020; Johnson and Mueller 2002; Morse et al. 2012; Salzberger et al. 2018;), a century health alert so to speak. The recent infectious pandemics, with an exception of the Flu pandemic of 2009, are all associated with coronaviruses. However, the magnitudes in terms of confirmed cases and mortality differ markedly. The SARS-CoV-2 pandemic of 2019 represents the overwhelming numbers of confirmed cases and mortality (Johns Hopkins University Coronavirus Resource Center, 2021); the MERS-CoV pandemic of 2012 represents the least number of confirmed cases but the highest mortality (Azhar et al. 2019; Chafekar and Fielding 2018); and the SARS-CoV pandemic of 2002 is in the middle (Anderson et al. 2004; Hui and Zumla 2019).

While vaccines are an important part of preventative measures to stop the spread, some coronaviruses have shown rapid adaptability and differentiation. As different strains are identified, the pathogen’s ability to mutate could outstrip our ability to create targeted vaccines. For instance, the new SARS-CoV-2 delta variant has shown vaccine breakthrough in Pfizer, Moderna, and Covaxin vaccines (https://pubmed.ncbi.nlm.nih.gov/34268529/). Therefore, it is necessary to have a treatment-based approach with anti-viral agents in addition to a preventative-based approach. The urgency to tackle the current pandemic in therapeutics has been focused largely on repurposing drugs for SARS-CoV-2. Remdesivir is originally developed for Ebola viral disease and has been shown to exert a broad-spectrum of viruses including coronaviruses (Eastman et al. 2020). Therefore, remdesivir is an ideal candidate to be repurposed for COVID-19.

## Overview of remdesivir

COVID-19 has become the biggest global health crisis in the modern history (Bassetto et al. 2021; Johns Hopkins University Coronavirus Resource Center, 2021;  Tabish 2020). This crisis is amplified by lack of specific therapeutics and high levels of transmission (Alshaeri and Natto 2020; Bassetto et al. 2021; Inglesby 2020; Linka et al. 2020; Liu et al. 2020; Song et al. 2020). SARS-CoV-2, the pathogen of COVID-19, has a basic reproduction number (R0 value) of 1.8–3.6 (Linka et al. 2020; Liu et al. 2020; Song et al. 2020). In certain regions, the R0 value goes as high as 5.0, pointing to extremely fast transmission (Linka et al. 2020; Liu et al. 2020; Song et al. 2020). Although SARS-CoV-2 belongs to the family of coronavirus like members of SARS-CoV and MERS-CoV, SARS-CoV-2 structurally differ markedly from other coronaviruses (Liya et al. 2020; Rabaan et al. 2020; Satarker and Nampoothiri 2020). As a result, existing anti-coronaviral agents are not effective (Shamsi et al. 2021). The urgency to tackle this pandemic in the area of therapeutics has been focused largely on repurposing drugs for SARS-CoV-2 (Chenoweth et al. 2020; Dong et al. 2020; Gao et al. 2020; Gordon et al. 2020; Gurwitz 2020; Ko et al. 2020; Lai et al. 2020; Martinez 2021; Shanmugaraj et al. 2020; Ton et al. 2020; Warren et al. 2016; Yao et al. 2020). Indeed, several antiviral agents targeting other viruses demonstrate reasonable efficacy such as the anti-Ebola agent remdesivir, the anti-HIV combination of lopinavir/ritonavir and the anti-parasitic drug *the anti-parasitic drug* avermectin (Bixler et al. 2017; Gilead Sciences 2020; NIH clinical trial NCT04280705 of remdesivir to treat COVID-19 begins 2020; Hoenen et al. 2019; Lo et al. 2017; Goldman et al. 2020; Grein et al. 2020; Siegel et al. 2017). Remdesivir appears to be the most promising (Beigel et al. 2020; Goldman et al. 2020; Grein et al. 2020; Spinner et al. 2020; Wang et al. 2020), and represents the first treatment for COVID-19 approved by the United State Food and Drug Administration (FDA News 2020). Remdesivir was initially granted for emergency use authorization and later for full approval.

### Chemical and structural features of remdesivir

**Fig. 1:**
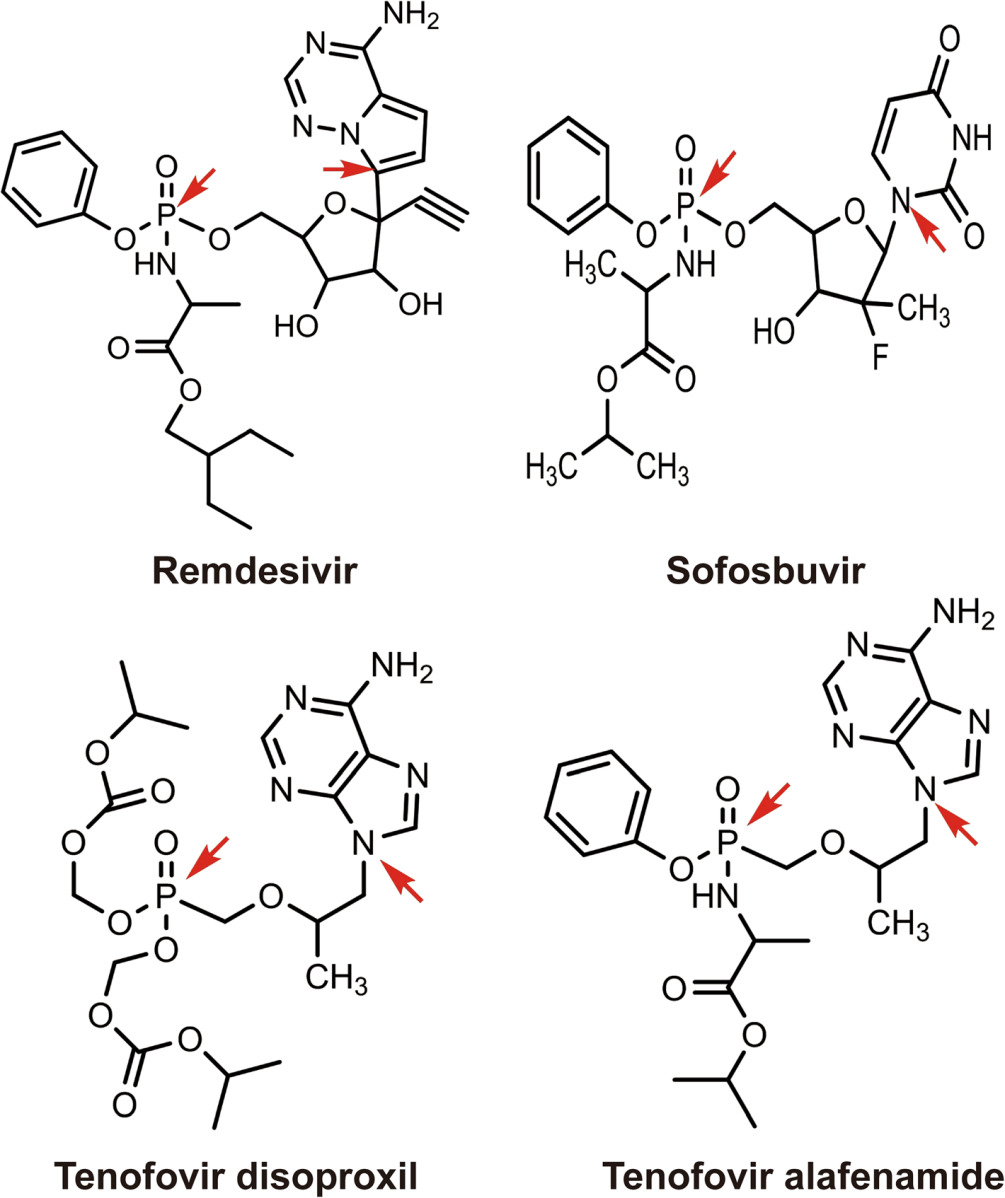
Chemical structure of remdesivir, sofosbuvir, tenofovir disoproxil and tenofovir alafenamide Those are nucleoside/nucleotide drugs featured by the heterocyclic ring linked to the phosphorus atom at the center. The connecting atoms are pointed by a red arrow

Remdesivir structurally belongs to the large class of nucleoside/nucleotide drugs (Cavaliere et al. 2017; LiverTox 2020; Meier 2017; Mirza 2019). Drugs in this class usually have anti-viral, anti-cancer and immunosuppressive activities (Borbone et al. 2021; Damaraju et al. 2003; Khungar and Han 2010; Krecmerova 2017; Stucker and Ackermann 2011). These drugs generally have a heterocyclic ring linked to the phosphorus atom at the center (Fig. 1, the connecting atoms marked with a red arrow). Interestingly, H-P linker (heterocyclic ring-phosphorus) varies among these therapeutics (Fig. 1). The anti-HIV drugs tenofovir disoproxil and tenofovir alafenamide, essential medicines listed by the World Health Organization (WHO), have a linker of a propane (Fig. 1), whereas remdesivir and the paradigm shift anti-hepatitis C viral agent sofosbuvir have a linker of oxolane (Santander-Ballestín et al. 2021; Tao et al. 2020). Even between sofosbuvir and remdesivir, the linker varies with strong implications of pharmacological activities (Fig. 1). Remdesivir but not sofosbuvir has a cyano structure attached to oxolane. The cyano structure is implicated in anticancer activity (Jordheim et al. 2013; Labbé et al. 2020; Liu et al. 2021; Ruchelman et al. 2011; Tretyakova et al. 2019). Finally, remdesivir, like others, is an ester and the ester linkage increases lipophilicity critical for cell permeability. This is particularly of significance as remdesivir has a relatively poor water-solubility (European Medicine Agency 2020).

### Mechanism of action

**Fig. 2:**
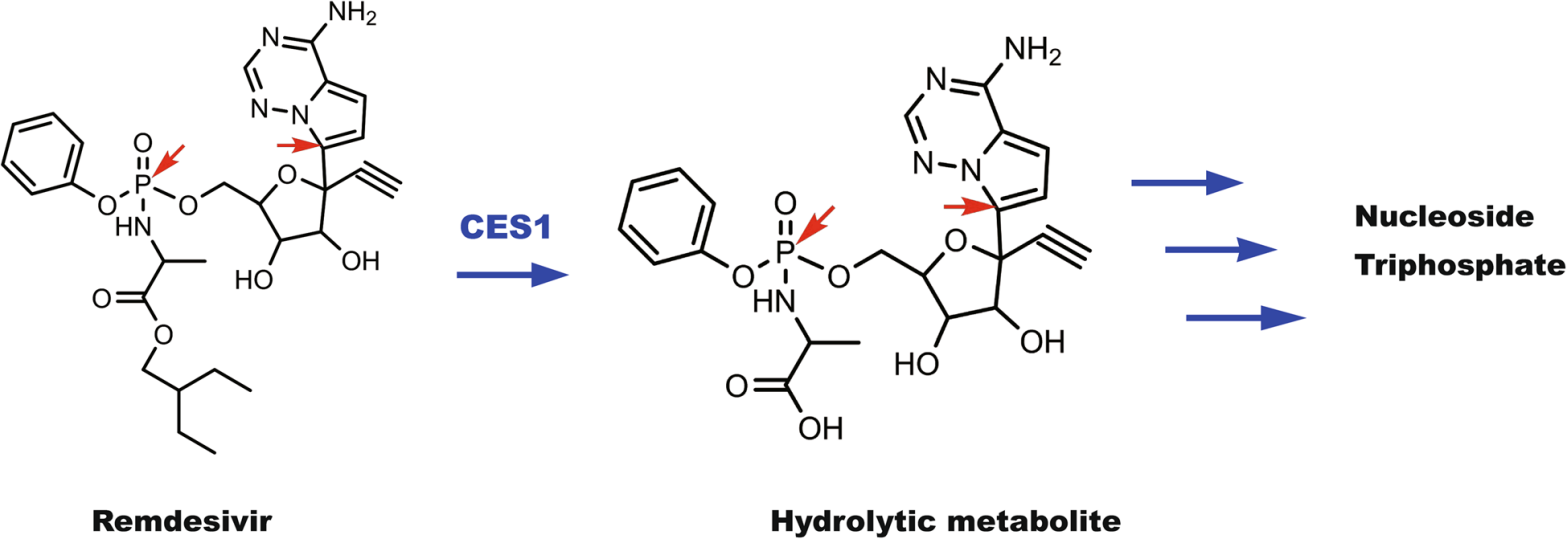
Therapeutic activation of remdesivir. This antiviral agent undergoes hydrolysis followed by several phosphorylation steps to form the antiviral metabolite nucleoside triphosphate. In human, the hydrolysis is achieved by CES1 but enzyme(s) for phosphorylation remains to be determined

Remdesivir undergoes hydrolysis initially followed by phosphorylation steps to form nucleoside triphosphate (Fig. 2) (Ottoni et al. 2020). It is the phosphorylated metabolite that delivers potent antiviral activity through distinct but related mechanisms (Fig. 2): (A) interfering with the action of viral RNA-dependent RNA polymerase (RdRp); (B) evading exoribonuclease-proofreading; and (C) causing delayed/cyano-group mediated chain termination of viral genome (Chen et al. 2020; Malin et al. 2020; Ottoni et al. 2020; Singh et al. 2020; Tchesnokov et al. 2019; Yin et al. 2020). We have shown that human carboxylesterase-1 (CES1), a highly efficient enzyme, was involved in the hydrolytic activation of remdesivir (Shen et al. 2021a, b). However, the precise identity of enzyme(s) for phosphorylation remain to be determined. As for the three mechanisms of action, it is clear that mechanisms A and C share the ultimate outcomes: delayed viral replication and in favor of antiviral activity. The mechanism of action B, on the other hand, can be considered as both desirable and non-desirable actions. Proofreading of genetic replications stabilizes the genome of virus but lack of strong proofreading capacity leads greater-than-expected instability of mutations. The emerged variants of SARS-CoV-2, with increased transmission capacity and even greater clinical severities, have argued that cautions must be exercised in this regard (Dicken et al. 2021; Martin et al. 2021).

### Broad spectrum of antiviral activity

As mentioned above, remdesivir was originally developed against Ebola viral infection (Bixler et al. 2017; Hoenen et al. 2019; Warren et al. 2016). Interestingly, this antiviral agent has since been shown to exert a broad spectrum of activity against as many as seven viral families (Jean et al. 2020; Pruijssers et al. 2020). These viruses, as specified in their genome, range from positive to negative, and to ambisense RNA viruses (Table 1). Critically, remdesivir has exhibited high potency towards these viruses with an exception of Hantaviridae. Members of the family Filoviridae are highly sensitive towards remdesivir with an EC_50_ value of as low as 3 nM (concentrations with half-maximal inhibition) (Table 1). Notable members in this family are Ebola virus and Marburg virus, which cause severe diseases known as viral hemorrhagic fevers (Reynolds and Marzi 2017; Shifflett and Marzi 2019). Majority of viruses from the family of Coronaviridae, which SARS-CoV-2 belongs to, are highly sensitive to remdesivir as well (Table 1) (Jean et al. 2020; Malin et al., 2020; Pruijssers et al. 2020). With human lung cells and primary human airway epithelial cultures, remdesivir inhibits SARS-CoV-2 replication with an EC_50_ value of 0.01 μM (Pruijssers et al. 2020). However, Coronaviridae members exhibit large strain differences from an EC_50_ of 0.02 to 4.90 μM (Table 1), representing an ~ 500-fold difference.

**Table 1:** Broad-spectrum antiviral activity

Family	Genome	Strains tested	EC_50_ (μM)^a^
Arenaviridae	Ambisense RNA	3	0.47–4.50
Coronaviridae	Positive RNA	20	0.02–4.90
Flaviviridae	Positive RNA	5	0.06–4.20
Filoviridae	Negative RNA	14	0.003–0.14
Hantaviridae	Negative RNA	1	7.00
Paramyxoviridae	Negative RNA	8	0.02–0.79
Pneumoviridae	Negative RNA	3	0.02–0.05

^a^EC_50_: Concentrations with half-maximal inhibition

## Efficacy and safety

COVID-19 has become the biggest global health crisis in the modern history, and its acceleration in a relatively short period presented unprecedented urgency (Johns Hopkins University Coronavirus Resource Center, 2021; Hu et al. 2021). The urgency has led to a strategy of repurposing of existing drugs as a viable and probably the most efficient approach to deal with COVID-19. Indeed, reasonable efficacy and safety profiles have been reported in relevance to this strategy not only for remdesivir but also for others (Bixler et al. 2017; Gilead Sciences 2020; NIH clinical trial of remdesivir to treat COVID-19 begins 2020; Hoenen et al. 2019; Lo et al. 2017; Goldman et al. 2020; Grein et al. 2020; Siegel et al. 2017). Among all of the repurposing medicines, remdesivir has been extensively studied. On the other hand, SARS-CoV-2 behaves differently from others such as SARS-CoV and MERS-CoV, two highly related viruses that have caused pandemics (Liya et al. 2020; Rabaan et al. 2020; Satarker and Nampoothiri 2020; Shamsi et al. 2021). Nevertheless, below is a brief discussion of remdesivir regarding efficacy, safety and potential mechanisms for safety concerns.

### Efficacy of remdesivir

The efficacy of remdesivir has been studied by several research identities: single research laboratories or multiple-institutional or even global efforts. The results are informative but not conclusive as many variables are involved in the study design and/or the primary outcomes of a study to pursue. Table 2 listed several remdesivir clinical trials and their efficacy outcomes. While there are some studies that support the use of remdesivir, the majority of studies conclude that there were no statistically significant clinical benefits. The Grein study found that 68% patients hospitalized for severe Covid-19 showed clinical improvement (Grein et al. 2020) and similarly, the Beigel study reported that remdesivir was superior to placebo in shortening the time to recovery and lowering respiratory tract infection (Beigel et al. 2020). Conversely, multiple studies have not found significant clinical improvement (Goldman et al. 2020; Wang et al. 2020), or difference in clinical status in moderate COVID-19 patients treated with remdesivir compared to regular standard of care (Spinner et al. 2020). The Solidarity study concludes that remdesivir has little or no effect on hospitalized patients with COVID-19, as indicated by overall mortality, initiation of ventilation, and duration of hospital stay (WHO Solidarity Trial Consortium 2021). It should be noted that the Solidarity study, not shown in Table 2, represented a global effort with out categorical details (WHO Solidarity Trial Consortium 2021).

**Table 2:** Efficacy of remdesivir in human clinical trials

Characteristic	^**a**^Beigel	^**b**^Wang	^**c**^Grein	^**d**^Goldman	^**e**^Spinner
Randomized controlled trial	Yes^1^	Yes^1^	No	Yes^2^	Yes^2^
Median time to recovery (Remdesivir)	10 days	21 days		11 day (10-day treatment)	
Median time to recovery (Control)	15 days	23 days		10 day (5-day treatment)	
Days to recovery (Remdesivir/control)	0.67	0.91		1.10	
Clinical improvement (10-day)			68%		65%
Clinical improvement (5-day)					70%

^a^ Beigel et al. 2020; ^b^ Wang et al. 2020; ^c^ Grein et al. 2020; ^d^ Goldman et al. 2020; ^e^ Spinner et al. 2020

^1^Randomized, double-blind, placebo-control clinical trials; ^2^randomized, open-label clinical trials

### Safety of remdesivir

The clinical studies, as discussed above, have pictured an encouraging but serious concerns regarding the use of remdesivir for COVID-19 (Table 3) (Beigel et al. 2020; Goldman et al. 2020; Grein et al. 2020; Spinner et al. 2020; Wang et al. 2020). There are many contributing factors to the conflicting observations including study design, patient populations, existing conditions, severity of COVID-19, use of other medications, and intrinsic adverse effects of remdesivir (discussed below). Indeed, the discontinued rate was as high as 11.6% (Table 3, The Wang study). In consistent with the high discontinued rates, the rates of serious adverse events were high as well (Beigel et al. 2020; Goldman et al. 2020; Grein et al. 2020; Spinner et al. 2020; Wang et al. 2020). Interestingly, remdesivir, when used for a longer duration such as 10 versus 5 day-treatment, caused greater number of adverse events (the Goldman study) or deaths (the Spinner study (Table 3). The serious adverse events range from cardiovascular events, to pulmonary disorders, and to hepatic concerns (Beigel et al. 2020; Goldman et al. 2020; Grein et al. 2020; Spinner et al. 2020; Wang et al. 2020). In terms of mortality, the results are not quite conclusive. Some studies have reported similar or comparable rates of death between remdesivir and control groups (Table 3) (Spinner et al. 2020; Wang et al. 2020).

**Table 3:** Safety of remdesivir in human clinical trials

Characteristic	^**a**^Beigel	^**b**^Wang	^**c**^Grein	^**d**^Goldman	^**e**^Spinner
Discontinued rate (Remdesivir) 10-day	9.8%	11.6%	7.5%	10.2%	4.1%
Discontinued rate (Remdesivir) 5-day				4.5%	2.1%
Discontinued rate (Control) 10-day	13.5%	5.1%			
Discontinued rate (Remdesivir/control)	0.73	2.27		2.27^f^	
Serious adverse events (Remdesivir-10 day)	24.6%	18.1%	22.6%	34.5%	5.2%
Serious adverse events (Remdesivir-5 day)				21.0%	4.7%
Serious adverse events (Control)	31.6%	25.6%			9.0%
Adverse events (Remdesivir/control)	0.78	0.69		1.64	
Death (Remdesivir-10 day)	10.9%	14.2%	13.2%	10.7%	1.6%
Death (Remdesivir-5 day)				8.0%	1.0%
Death (Control)	14.8%	12.8%			2.0%

^a^ Beigel et al. 2020; ^b^ Wang et al. 2020; ^c^ Grein et al. 2020; ^d^ Goldman et al. 2020; ^e^ Spinner et al. 2020

^f^ Comparison between 10-day and 5-day group

### Mechanistic links to the safety concerns

The safety concerns of remdesivir are likely resulted from several important mechanisms. First, COVID-19 is a disease with multiple phases, typically from the initial infectious phase, to viral replication phase and to pathological phase. The phase-symptoms are clinically defined as mild, pulmonary and inflammatory stage (Soy et al. 2020). As a result, remdesivir likely delivers clinical benefits depending on a stage of the disease. Second, COVID-19 impacts functions of multiple organs. While the respiratory system is considered to be the primary route for infectious transmission, there are other systems such as the digestive system identified to play such as role (Gavriatopoulou et al. 2020). Nevertheless, the relatively pathological impact among these organs, once again, may vary depending on a disease stage. And third, COVID-19 patients usually receive diverse types of therapeutic approaches such as oxygen therapy, anti-inflammatory therapy, and of course antiviral therapy (Beigel et al. 2020; Goldman et al. 2020; Grein et al. 2020; Spinner et al. 2020; Wang et al. 2020).

**Fig. 3:**
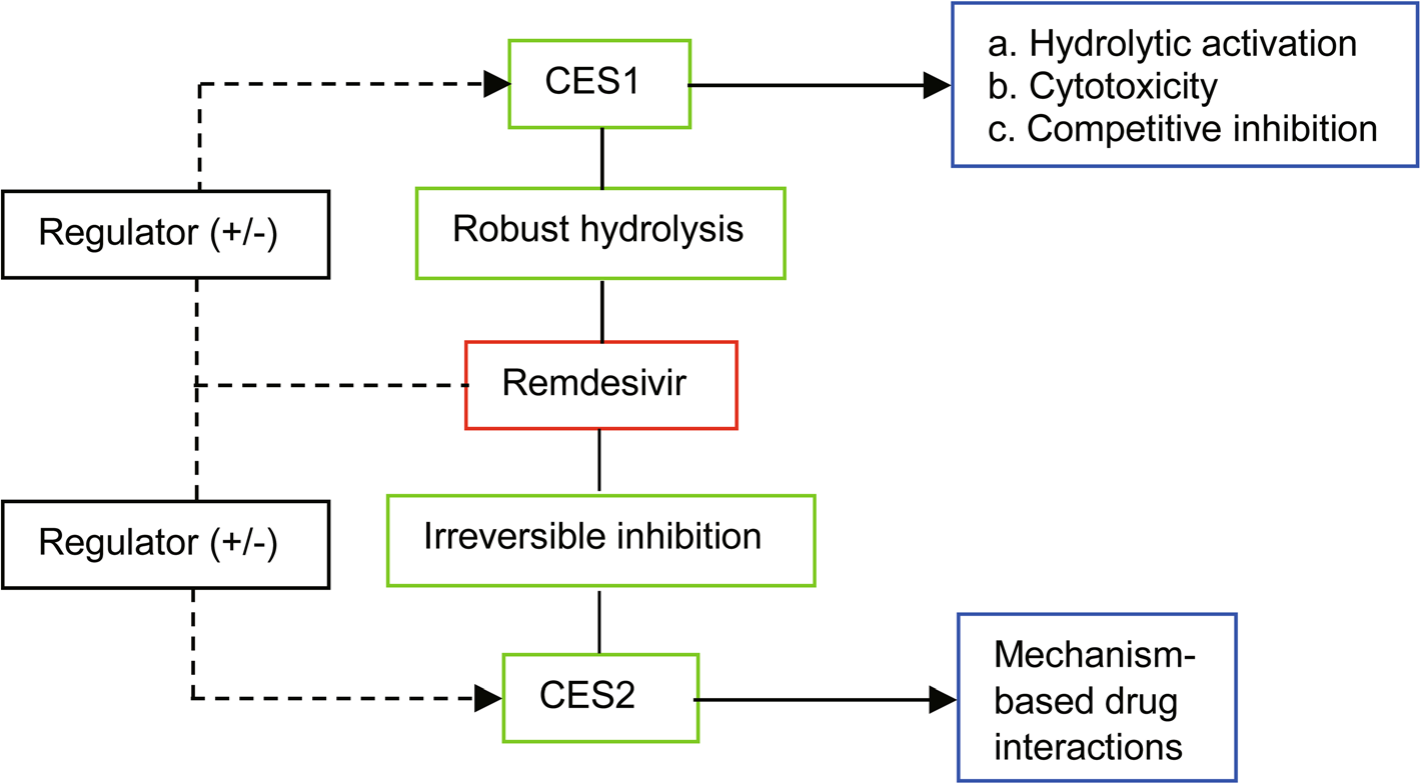
Diagrammatic presentation of carboxylesterases-based drug interactions with remdesivir. This virial agent is a substrate of CES1 but an irreversible inhibitor of CES2. Therefore, remdesivir catalytically impacts both enzymes (solid lines). In addition, many drugs and other xenobiotic compounds as well as disease mediators such as cytokines are established to regulate the expression of both enzymes. It is assumed that remdesivir has a potential of interacting with those factors (dotted lines)

The implications of drug-drug interactions and hepatic toxicity in the safety concerns point to an intimate involvement of remdesivir metabolism. Indeed, COVID-19 patients, probably in all cases, receive more than one or even more drugs (Beigel et al. 2020; Goldman et al. 2020; Grein et al. 2020; Spinner et al. 2020; Wang et al. 2020). Remdesivir is an ester prodrug and it is therefore assumed to have hydrolysis-based interactions. Even for hydrolytic interactions, the underlying mechanisms can be distinct with two notable actions: regulated expression of remdesivir hydrolase(s) (dotted lines) and modulated catalysis toward remdesivir (solid lines) (Fig. 3). The modulated catalysis toward remdesivir hydrolysis is considered to be intrinsic as hydrolysis is required for the therapeutic activity of remdesivir. It has been confirmed that remdesivir was hydrolytically activated by CES1 (Shen et al. 2021a). However, excessive hydrolysis causes severe cytotoxicity dominantly through inhibited proliferation and enhanced apoptosis (Shen et al. 2021a). In addition, remdesivir has been shown to irreversibly inhibit carboxylesterase-2 (CES2) (Shen et al. 2021b). This carboxylesterase is a major hydrolase with distinct substrate specificity, regulated expression and tissue distribution (Chen et al. 2012; Shen et al. 2019; Shen and Yan 2017; Shi et al. 2006; Shi et al. 2008; Tang et al. 2006; Xiao et al. 2012; Yang et al. 2007; Yang et al. 2009; Zhu et al. 2000). Conceivably, irreversible inhibition of this hydrolase is a contributing factor to drug-drug interactions with potential pharmacological and toxicological significance.

## COVID-19 animal models

Animal models are critical for pathological understanding and therapeutic confirmation. During the past year and half, concerted efforts have been made in developing animal models for COVID-19 (Cleary et al. 2020; Ludwig and Zarbock 2020; Muñoz-Fontela et al. 2020; Veenhuis and Zeiss 2021; Zeiss et al. 2021). These efforts have been focused on several critical aspects: transmissibility, disease process, therapeutic efficacy, immune response, and species-differential mechanisms. Studies on the transmissibility have been focused on ACE2 receptor (angiotensin-converting enzyme-2) (Bao et al. 2020; Shang et al. 2020; Sun et al. 2020), a dual functional protein as a receptor and an enzyme critical for physiological function (i.e., blood pressure) and infectious transmissibility (i.e., SARS-CoV-2). These studies have firmly established the critical role of ACE2 in the transmissibility. Animals sharing with humans the higher ACE2 sequence identity, generally have higher rates of transmissibility (Chan et al. 2020). The most commonly used mammalian research model mouse, sharing relatively a low identity with human in terms of ACE2 sequence, does not confer efficient transmission of SARS-CoV-2 (Muñoz-Fontela et al. 2020; Zeiss et al. 2021). Nevertheless, efforts have successfully created various mouse COVID-19 models by genetic approaches (Muñoz-Fontela et al. 2020; Zeiss et al. 2021), such as replacement of the mouse *ace2* with the corresponding human *ACE2*. It should be emphasized that mutations of the receptor binding domain of SARS-CoV-2 is recognized to be critical for increased transmissibility and even increased morbidity and mortality (Greaney et al. 2021; Jackson et al. 2021; Leung et al. 2020; Guruprasad 2021).

### Disease modeling of COVID-19

While transmissibility is the determinant factor for the passage of infectious diseases, pathological changes, in line with the process, is one of the most, probably the most important factor for modeling. Table 4 shows major COVID-19 animal models with specifics of viral replication, clinical signs and immune responses (Muñoz-Fontela et al. 2020; Zeiss et al. 2021; Veenhuis and Zeiss 2021). These categories or manifestations are commonly seen among COVID-19 patients (Zeiss et al. 2021). However, not all information on these categories has been collected among these animal models. Nevertheless, Syrian hamsters model well to humans (Table 4). Pigs, chickens and ducks are not susceptible to COVID-19 and not viable animal candidates (Bao et al. 2020; Imai et al. 2020; Lakdawala and Menachery 2020). Dogs have a low susceptibility to SARS-CoV-2. Infectious viral RNA was not detected in pharyngeal swabs of inoculated dogs, and four of the six dogs failed to seroconvert (Shi et al. 2020). Coronaviruses are endemic among bats, and there is a bat SARS-like CoV strain that shares a common ancestor with SARS-CoV-2, diverging approximately 40–70 years ago (Boni et al. 2020). Bats inoculated with SARS-CoV-2 displayed high viral loads and live virus could be obtained from oral swabs, trachea and nasal epithelium. However, bats do not display any clinical signs of infection, giving credence to their known viral tolerance. Compared to ferrets, their antibody response is less robust (Schlottau et al. 2020).

**Table 4:** Major SARS-CoV-2 animal models and reported manifestations

Manifestations	Adapted mice^a^	Cat	Ferret	Hamster	Non-human primates
Viral shedding	√	√	√	√	√
Fever/nasal discharge/labored breathing	√		√	√	
Pneumonia	√	√		√	√
Gastrointestinal/renal signs				√	
Cardiovascular/neurological signs	√			√	
Sex-difference in clinical signs				√	
Aging–related severity/susceptibility	√			√	√
Elevated systemic inflammation	√			√	√
Innate immunity	√		√	√	√
T cell response	√		√		√
B cell response	√		√	√	√

^a^Various types of genetically modified mice with differential manifestations

### Efficacy of remdesivir in animal models

Therapeutic or efficacy confirmation is another major step, probably the most critical step in terms of managing a disease (Johansen et al. 2020; Sheahan et al. 2020; Yu et al. 2020). Modeling of therapeutic confirmation, compared with disease modeling itself, is complicated by the interplay between host and therapeutic agent, remdesivir in this case. Nevertheless, there are several studies in the literature about efficacy of remdesivir against SARS-CoV-2 (Martinez et al. 2021; Pruijssers et al. 2020; Williamson et al. 2020; Ye et al. 2021; Yuan et al. 2021). These studies are informative but the information is incomplete and/or inconsistent in terms of study design, dosage regimens, and/or the defined outcomes. For example, the dosage regimens were different and so were the dosing routes in some cases. Table 5 summarized the results from these studies on the therapeutic confirmation.

**Table 5:** Efficacy of remdesivir in SARS-CoV-2 animal models

Author	Model	L/M dose	Route	Vial burden	Clinical improvement
Pruijssers et al. 2020	Mouse^a^	25 mg/kg^b^	sc	> 99 ↓	↑↑↑
Williamson et al. 2020	R. macaques	10/5 mg/kg/d	iv	100 x ↓	↑↑↑↑
Ye et al. 2021	Hamster	15 mg/kg	ip	> 80% ↓	↑↑↑↑
Yuan et al. 2021	Hamster	15 mg/kg	ip	~ 20% ↓	↑↑

^a^ Ces1c knockout; ^b^ Twice a day; *R* rhesus, *sc subcutaneous injection, iv i*ntravenous injection, *ip* intraperitoneal injection

Pruijssers et al. investigated the efficacy of remdesivir in a mouse model (Ces1c knockout) (Pruijssers et al. 2020). The animals were inoculated with a chimeric virus (SARS1/SARS2-RdRp). This chimeric virus encodes the RNA-dependent RNA polymerase of SARS-CoV-2. Remdesivir treatment was initiated at 1-day post inoculation (1 dpi) at 25 mg/kg through subcutaneous injection and continued every 12 h until the end of the study at 5 dpi. The viral burden was decreased by at least 99% in the remdesivir group. Clinical signs such as lung hemorrhage and pulmonary function were drastically improved. Williamson et al. investigated the efficacy of remdesivir in a rhesus macaque model (Williamson et al. 2020). The treatment was initiated at 12 h after SARS-CoV-2 inoculation and continued once daily through 6 dpi. One group intravenously received a loading dose of 10 mg/kg remdesivir, followed by a daily maintenance dose of 5 mg/kg, and the other group received vehicle control. They reported that macaques treated with remdesivir did not show signs of respiratory disease with overwhelming reductions of viral burden. At necropsy, remdesivir-treated animals had lower lung viral loads and reduced lung damage. Thus, treatment with remdesivir initiated early during infection had a clinical benefit in rhesus macaques infected with SARS-CoV-2.

Ye et al. investigated the efficacy of remdesivir in hamsters through intraperitoneal injection (Ye et al. 2021). The treatment with remdesivir (15 mg/kg) was performed at 2 dpi and 3 dpi post-inoculation. The viral burden was monitored at 4 and 14 dpi as well as body weight daily. Remdesivir reduced the viral burden in multiple respiratory tissues (e.g., nasal) by at least 80%. The body weight in remdesivir but not the vehicle group continued to increase. Promisingly, these parameters were improved further at 14 dpi. Yuan et al. investigated the efficacy of clofazimine (a leprosy medicine) in hamsters against SARS-C0V-2 infection (Yuan et al. 2021). Remdesivir was included as a positive control. The treatment with remdesivir (15 mg/kg) was performed at 1, 2 and 3 dpi through intraperitoneal injection. The viral burden was monitored at 4 dpi and body weight daily. Remdesivir reduced the viral burden by ~ 20% in the lung tissue. It should be noted that viral titers were determined by plaque-forming assay in the Yuan study (2021), whereas the Ye study used RT-PCR assay (Ye et al. 2021). The body weight of the remdesivir group was higher than that of the control group at 3 and 4 dpi (Yuan et al. 2021).

### Interspecies significance

The efficacy studies about remdesivir in animal models are informative but may not recapitulate clinical settings completely (Martinez et al. 2021; Pruijssers et al. 2020; Williamson et al. 2020; Ye et al. 2021; Yuan et al. 2021). It is encouraging that all of the studies have demonstrated benefits from the use of remdesivir, however, such a conclusion cannot be convincingly drawn from human clinical studies (Beigel et al. 2020; Goldman et al. 2020; Grein et al. 2020; Spinner et al. 2020; Wang et al. 2020). There are nonetheless several contributing factors on the study design. First, remdesivir treatment in the animal models was initiated 12 or 24 h after SARS-CoV-2 exposure. It is not clear whether this represents the situation in human clinical trials. Second, COVID-19 patients are generally treated with a loading dose of 200 mg with 9-day maintenance dose of 100 mg. It is not clear how closely the exposure of remdesivir in the animal models was in line with the human exposure. And third, there is a relatively large range in the dosage regimens among these models, and it was difficulty to draw a dosing-dependent efficacy. For example, the Pruijssers study used a daily dose of 50 mg (Martinez et al. 2021; Pruijssers et al. 2020; Williamson et al. 2020; Ye et al. 2021; Yuan et al. 2021), representing 3–10 times of the maintenance daily dose in the other studies. It was complicated even more that none of these studies fully monitored the metabolic fate of remdesivir (Martinez et al. 2021; Pruijssers et al. 2020; Williamson et al. 2020; Ye et al. 2021; Yuan et al. 2021).

**Fig. 4:**
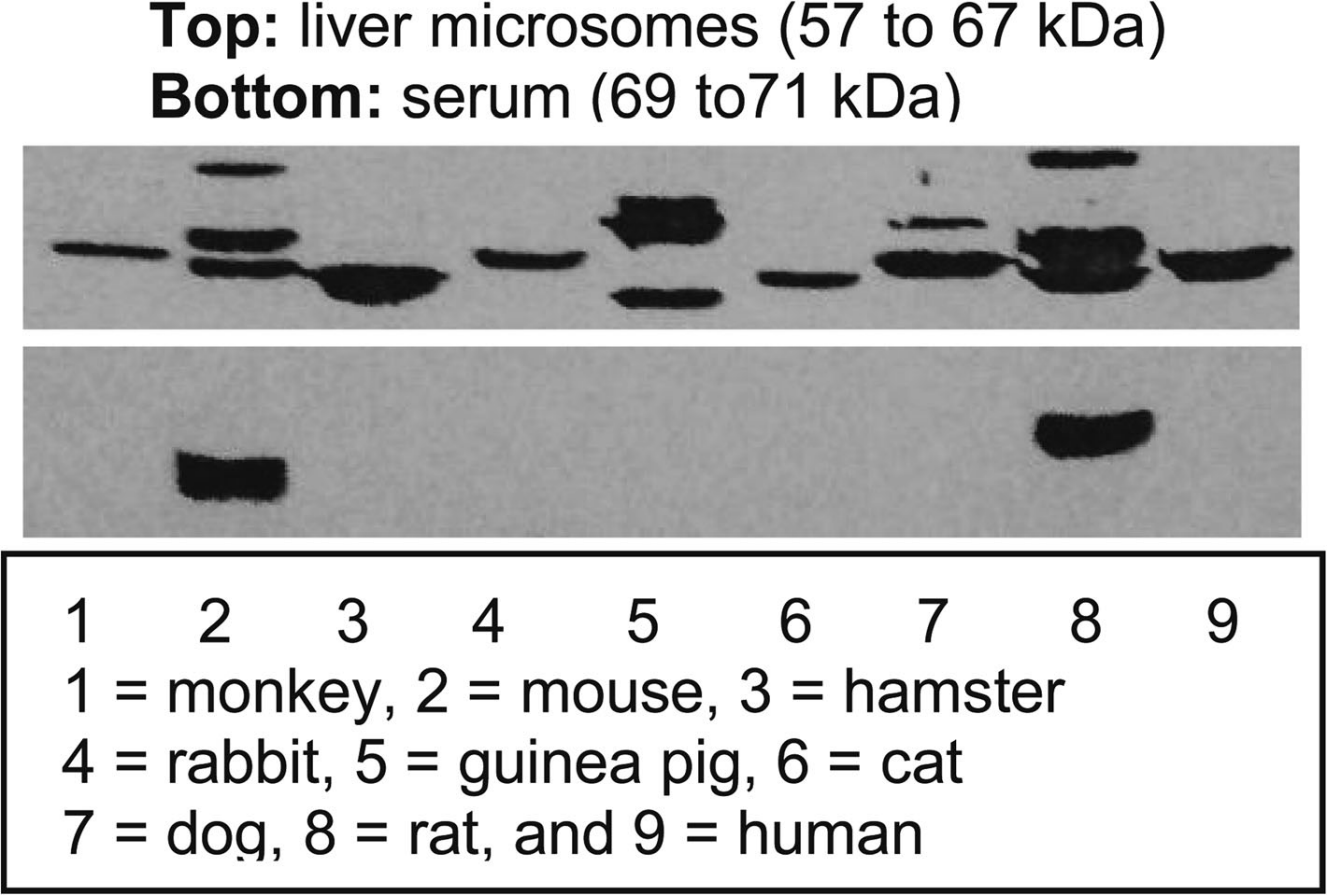
Immunoblots of liver microsomes and serum from various species with anti-rat Ces1d. Microsomes (10 μg) or serum (0.5 μL) from mature males were resolved by 7.5% SDS-PAGE and transferred electrophoretically to nitrocellulose membranes. The blots were blocked by milk and detected by the antibody against recombinant rat Ces1d through *E. coli* expression system. This antibody has been shown to have a broad-cross reactivity activity among carboxylesterases

Remdesivir is an ester/phosphorylation prodrug and hydrolysis of the ester represents the first step toward the therapeutic activation (Ottoni et al. 2020). Next we examined whether the commonly used COVID-19 animal models have comparable expression of carboxylesterases, a highly efficient class of hydrolases. we performed a Western analysis with liver microsomes and serum from 9 different species with the antibody against rat Ces1d. This antibody was raised against bacterially expressed Ces1d. No glycosylation has been shown to cross-react with any carboxylesterase (Xiao et al. 2012; Yan et al. 1995). As showed in Fig. 4, this antibody recognized a single band in the liver microsomes from monkey, hamster, rabbit, cat and human but multiple bands in others such as mouse, guinea pig, and dog. The intensity of the band varied from one to another species. For example, the intensity varied by at least 3-fold between monkey and hamster. In addition, both mice and rats expressed high levels of serum carboxylesterase (Fig. 4). These findings conclude that cautions must be exercised regarding ester drugs in terms of their pharmacodynamics and pharmacokinetic determinants among various species.

## Cross-species therapeutic potentials beyond SARS-CoV-2

Remdesivir exerts a broad spectrum of activity against as many as seven viral families with high potency (Table 1) (Jean et al. 2020; Malin et al., 2020; Pruijssers et al. 2020). In addition to differences in the genome, these viruses differ in organ and tissue tropisms. Table 6 showed some examples of remdesivir or its precursor (GS-441524) as a potent therapeutic against these viruses. All of the examples have in vivo data with an exception of the porcine epidemic diarrhea virus, PEDV). This virus causes acute diarrhea in neonatal piglets with high mortality (de Wit et al. 2020; Dickinson et al. 2020; Lo et al. 2019; Mulangu et al. 2019; Murphy et al. 2018; Paltrinieri et al. 2020; Pedersen et al. 2019; Porter et al. 2020; Xie et al. 2021; Yin et al. 2021). Importantly, PEDV and SARS-CoV-2, belong to the Coronaviridae family. Interestingly, vaccines prepared from SARS-CoV-2 fusion protein have been shown to protect against PEDV infection (Xie et al. 2021), pointing to immunological cross-protection. This is of significance as this phenomenon provides immunological basis to control future epidemic (pandemic) by those that are immunologically related to previous epidemic/pandemic pathogens.

**Table 6:** Examples of remdesivir in treating other viral diseases

Human or animals	Virus	Viral family	Mortality	Viral replication	Clinical improvement	Cell culture
^a^Pig	PEDA^1^	Coronaviridae				EC_50_: 0.74 μM
^b^Human trial b	Ebola	Filoviridae	53%^2^			
^cdef^Cat	FIP	Coronaviridae	83.9%^3^, 0%^4^	> 99%↓	↑↑↑↑	
^g^Macaque	Marburg	Filoviridae	17%	↓↓↓	↑↑↑	
^h^Macaque	MERS-CoV	Coronaviridae		↓↓↓↓	↑↑↑↑	
^i^Monkeyi	Nipah	Paramyxoviridae	0%	↓↓	↑↑↑↑	
^j^Mousek	SARS-CoV	Coronaviridae		> 99%↓	> 50% ↑	

^1^Porcine epidemic diarrhea virus; ^2^No placebo control but worsening compared with ZMapp treatment group (positive control); ^3^No treatment control as this was naturally occurring infection; ^4^ Experimmental infection; ^3,4^The parent drug of remdesivir (GS-441524) for the treatment

^a^Xie et al. 2021; ^b^Mulangu et al. 2019; ^c^Dickinson et al. 2020; ^d^Murphy et al. 2018; ^e^Pedersen et al. 2019; ^f^Yin et al. 2021; ^g^Porter et al. 2020; ^h^ de Wit et al. 2020; ^i^Lo et al. 2019; ^j^Johansen et al. 2020

### Efficacy of remdesivir against Ebola virus disease

Remdesivir was originally developed against Ebola viral infection (Mulangu et al. 2019), and *in vitro* studies have reported high potency with an EC_50_ value within low nanomolar level (Jean et al. 2020; Pruijssers et al. 2020). A randomized controlled trial on the treatment was carried out with remdesivir, along with three monoclonal preparations (Mulangu et al. 2019). All patients received standard care and were randomly assigned in a 1:1:1:1 ratio to intravenous administration of antibody ZMapp, remdesivir, single monoclonal antibody MAb114, or triple monoclonal antibody REGN-EB3. Remdesivir was given intravenously at a loading dose on day 1 (200 mg in adults, and adjusted for body weight in pediatric patients), and a daily maintenance dose (100 mg in adults) thereafter for 9 to 13 days. Compared with other treatment groups, the remdesivir group had the highest mortality rate (53.1%). Among children at an age of 5 years, the mortality rate reached as high as 62.5%. Clearly, the monotherapy of remdesivir was not superior to other therapeutics. Nevertheless, this study did not consider factors such as disease stage, population differences, and potential drug-drug interactions (Mulangu et al. 2019).

### Protection of remdesivir against Nipah virus

Nipah virus (NiV) is an RNA virus that belongs to the family of Paramyxoviridae, a family of negative-strand RNA viruses (Lo et al. 2019). The reservoir of NiV is the *Pteropus* fruit bat and likely gained transmission to humans through pigs. It was first identified in Malaysia in 1998 and has since caused numerous outbreaks in and around South and Southeast Asia (Hauser et al. 2021; Rathish and Vaishnani 2021). The mortality rate of NiV reaches as high 75%. There are no vaccines available for this deadly virus. It is a priority pathogen of the WHO due to its propensity for causing outbreaks. Members of the Paramyxoviridae family are highly sensitive to remdesivir (Table 1). An *in vivo* study was carried out in Africa green monkeys (Lo et al. 2019). Animals were intratracheally inoculated with NiV. Remdesivir treatment was initiated 24 h after the inoculation at 10 mg/kg through intravenous infusion. The treatment was continued once daily for 12 days. All control animals developed severe respiratory disease signs and were euthanized 7 or 8 dpi due to the disease severity (humane endpoints) (Lo et al. 2019). In contrast, none of remdesivir-treated animals developed severe symptoms. This study concluded that remdesivir represented a promising antiviral treatment for NiV infection.

### GS-441524, the parent drug of remdesivir for natural or experimental feline infectious peritonitis

Feline infectious peritonitis (FIP), a deadly disease for domestic cats, is caused by FIP virus (FIPV), probably by FIPV-related viruses as well (Dickinson et al. 2020; Murphy et al. 2018; Paltrinieri et al. 2020; Pedersen et al. 2019; Yin et al. 2021). FIPV and SARS-CoV-2 share several major traits: (A) they belong to the Coronaviridae family and (B) both FIPV and SARS-CoV-2 have high transmissibility although the former is more deadly. On the other hand, they differ in organ tropism: FIPV targets predominantly the gastrointestinal tract, whereas SARS-CoV-2 targets predominantly the pulmonary system (Dickinson et al. 2020; Gavriatopoulou et al. 2020; Murphy et al. 2018; Pedersen et al. 2019; Yin et al. 2021). Nevertheless, several investigators tested GS-441524, the parent drug of remdesivir, for the efficacy against natural and experimental FIPV infection (Dickinson et al. 2020; Murphy et al. 2018; Pedersen et al. 2019; Yin et al. 2021). Dickinson et al. treated four naturally occurring FIP cases with neurological manifestations and demonstrated clear clinical improvement (Dickinson et al. 2020). Yin et al. reported that FIP-suspected cats had a mortality rate of 67%, however, an overwhelming majority of cats treated with GS-441524 survived (Yin et al. 2021). Similar efficacy was reported by Pedersen (Pedersen et al. 2019). Finally, Murphy et al. reported in experimentally FIPV infected cats that GS-441524 caused a rapid and efficient reversal of clinical signs and returned to normality among all cats (Murphy et al. 2018).

## Conclusions/further perspectives

The COVID-19 pandemic is unprecedented even in the historical term and the unprecedentedness is featured by multiple surges, rapid identification of therapeutic options and accelerated development of vaccines. The therapeutic options have been focused largely on repurposing existing medicines. Remdesivir, originally developed for Ebola viral disease, is the first treatment of COVID-19 approved by the United States FDA. *In vitro* and animal studies have shown that this antiviral agent had broad-spectrum activities with high potency. However, human clinical trials for COVID-19 or Ebola have failed to confirm the favorable properties on both efficacy and safety from preclinical studies. One explanation is that animal models have not faithfully recapitulated the pathological and pharmacological processes in human. Another explanation is that COVID-19 patients in the trials have received multiple therapeutics with increased risk for drug-drug interactions. These interactions likely have profound-species differences. Finally, remdesivir requires hydrolysis and phosphorylation to exert antiviral activity and excessive hydrolysis increases cytotoxicity. Several options should be considered: (1) remdesivir is given through intravenous injection, a route that quickly builds high concentrations, and other administration routes should be considered to prevent concentration spikes that cause safety concerns; (2) formulations of remdesivir are so developed to minimize the toxicological potentials; and (3) the chemical structure of remdesivir, particular the ester linkage, should be modified to reduce the risk. Once again, remdesivir has been shown to be broad-spectrum and high potency. Optimization of administration routes, delivery formulations and chemical structure (e.g., the ester linkage) will signify not only for the current COVID-19 pandemic but also for future ones caused by remdesivir-sensitive viruses.

## Data Availability

This is a review article. The data supporting their findings can be found in the literature as described below.

## Abbreviations

ACE2: Angiotensin-converting enzyme 2
CES1: Carboxylesterase-1
Ces1d: Carboxyleseerase-1d
CES2: Carboxylesterase-2
COVID-19: Coronavirus disease 2019
EC_50_: Half maximal effective concentration
FIPV: Feline infectious peritonitis
MERS-CoV: Middle East respiratory syndrome coronavirus
NiV: Nipah virus
PEDV: Porcine epidemic diarrhea virus
SARS-CoV: Severe acute respiratory syndrome coronavirus
SARS-CoV-2: Severe acute respiratory syndrome coronavirus 2
